# Weight changes in hypertensive patients with phlegm-dampness syndrome: an integrated proteomics and metabolomics approach

**DOI:** 10.1186/s13020-021-00462-x

**Published:** 2021-07-07

**Authors:** Chi Zhang, Li Li, Shiping Cheng, Debajyoti Chowdhury, Yong Tan, Xinru Liu, Ning Zhao, Xiaojuan He, Miao Jiang, Cheng Lu, Aiping Lyu

**Affiliations:** 1 Dongzhimen Hospital, Beijing University of Chinese Medicine, Dongcheng District, Beijing, 100700 China; 2 Institute for Brain Disorders, Beijing University of Chinese Medicine, Beijing, China; 3 Institute of Basic Research in Clinical Medicine, China Academy of Chinese Medical Sciences, Beijing, China; 4 School of Nursing, Jiangxi University of Traditional Chinese Medicine, Nanchang, China; 5 Institute of Integrated Bioinformedicine & Translational Science, Hong Kong Baptist University, Kowloon Tong, Hong Kong, China; 6 School of Pharmacy, Second Military Medical University, Shanghai, China; 7 School of Chinese Medicine, Hong Kong Baptist University, Kowloon Tong, Hong Kong, China

**Keywords:** Hypertension, Chinese medicine syndrome, Metabolomics, Proteomics, Longitudinal study

## Abstract

**Background:**

Hypertension (HTN) patients who have phlegm-dampness syndrome (PDS) tend to be obese and have worse outcomes. However, the association of body weight (BW) changes and mechanisms underlying the pathophysiology of HTN-PDS are not well elucidated. This study aims to identify the longitudinal observations associated with the circulating markers discriminating BW changes of individuals with HTN-PDS.

**Methods:**

An integrative approach relying on metabolomics and proteomics was applied to serum samples from HTN-PDS patients in a prospective cohort to identify the plausible mechanistic pathways underpinning HTN-PDS pathophysiology. Study participants were determined to have experienced a weight change if they showed a 5%–15% increase/reduction in BW at the end of the follow-up period. The joint pathway analysis and network analysis were performed using Ingenuity Pathway Analysis (IPA®) on the serum samples obtained from the participants over the period.

**Results:**

The study involved 22 HTN-PDS patients who were overweight initially and were able to lose enough weight and 24 HTN-PDS individuals who developed overweight from normal BMI during a one-year follow-up. Our analysis suggested three types of phosphatidylcholine (PC) were altered. PC (22:2(13Z,16Z)/24:1(15Z)) and LysoPC (16:1(9Z)) were decreased in Queryweight gain samples, whereas the levels of PC (14:0/16:0) were increased in weight loss samples. The metabolomic analysis suggested 24 metabolites associated with HTN-PDS. Of them, 13 were up-regulated and 11 were down-regulated. The two-dimensional difference gel electrophoresis (2D DIGE) identified 45 phosphorylated proteins got altered in the HTN-PDS patients, wherein 23 were up-regulated and 22 were down-regulated. Integrated proteomic and metabolomics analyse acknowledged biomarkers PC, Complement C3, C4a/C4b, A2M and SERPINF1 as strong predictors for BW changes in HTN-PDS patients.

**Conclusion:**

The combined serum proteomic and metabolomic profiling reveals a link between BW change and the complement system activity, altered phosphatidylcholine metabolism in HTN-PDS patients. Future studies with larger cohorts are required to strengthen and validate these findings.

## Background

Hypertension (HTN) is one of the most important vascular risk factors worldwide [1, 2]. The prevalence and incidence of HTN are increasing in all countries and contribute to the global burden of cardiovascular diseases [3]. Individuals with HTN are usually treated similarly, but prognoses differ and are often poorly understood [4]. In most cases amid clinical settings, those patients used to receive pharmacological treatments with certain types of antihypertensive drugs such as β-Blockers, ACE inhibitors, angiotensin receptor blockers, calcium channel blockers, thiazide and thiazide-type diuretics, aldosterone antagonists, direct renin inhibitors [4]. Overweight and obesity are well-established risk factors associated with the development of HTN [5, 6]. They are also significantly associated with poor prognosis and increased complications [7, 8]. Weight loss is recommended as an effective nonpharmacologic approach for lowering blood pressure (BP) [1]. Clinical guidelines provide recommendations for maintaining a normal BW for the management of HTN [3, 6]. Numerous epidemiologic studies and interventional trials have also demonstrated the major role of weight change in determining HTN risk [2, 9–12].

Chinese medicine (CM) categorizes HTN as internal disease symptoms. Syndrome differentiation (SD) is a comprehensive analysis of clinical information gained by the diagnostic CM procedures [13]. As previously reported, phlegm-dampness syndrome (PDS) is a common syndrome associated with HTN [14–16]. Phlegm-dampness syndrome (PDS) is a syndrome that arises when dampness gathers to form phlegm, with different manifestations according to location: cough with profuse whitish expectoration and oppression in chest if phlegm obstructs the lung, and epigastric stuffiness, reduced food intake, and greasy taste in the mouth if phlegm accumulates in the spleen [17]. PDS individuals tend to be obese and have worse cardiac and vascular outcomes [15, 18, 19]. A mathematical model with more than 18,800 cases showed that compared with balanced constitution, the risk of obesity was also significantly higher with an OR of 4.34 and 95% CI of 3.52–5.36. Studies on the PDS are significantly important for the prediction and prevention of related diseases and maintenance of human health [14]. Transforming phlegm and eliminating dampness by weight management has been recommended [19, 20]. However, few studies have compared the impact of weight changes in opposite directions in HTN patients with PDS, although this reversible factor provides an opportunity to control HTN and its vascular consequences.

During the development of HTN, tissues and organs undergo structural and functional changes those are characterized by specific alterations in the type and amount of various proteins expressed [21]. Few studies relying on proteomic approach have exposed a wide range of proteins expressed in serum samples [22, 23]. Hence, characterizing their expressions and differential fluctuations in parallel with the development and progressions of HTN must edify to distinguish some protruding pathophysiological pathways [22, 23]. As the protein levels influence the metabolic profile, in return, the concentrations of the metabolites may affect protein expressions [24]. The physiological activity of HTN-PDS is complex, as using a single omics makes it hard to fully reveal the mechanism. Therefore, combining such wide information within the scope of systems biology approach deployed with human samples must enlighten more comprehensive understanding of the deep biology and pathophysiological processes of HTN [25, 26].

Here, in this longitudinal study, we have studied the metabolic changes in serum samples following exposure to BW changes throughout 1-year in individuals with HTN-PDS (*n* = 46, weight gain = 24, weight loss = 22) using an integrated proteomics and metabolomics analysis. The superiority of our approach is contented by its concurrent utilization of those omics techniques in determining the plausible biomarkers altered while BW changes and get reflected in the cohort of HTN patients longitudinally. It also ameliorated some of the deficiencies of each -omics technique when employed by itself. These markers may serve as potential therapeutic targets to manage HTN in clinical practice.

## Materials and methods

### Participants

A total of 5638 individuals (1556 females, 27.6%) undertook an annual health check-up at a community hospital in Beijing, China in 2016 and 2017. The inclusion criteria include the following: (1) participants were from a normal BMI to a high BMI (N–H) or from a high BMI to a normal BMI (H–N) as determined by standardized measurements described below, (2) patients must have HTN, PDS [27] and be without any active cardiovascular diseases, (3) patients must be aged ≥ 18 years at the beginning of the study, (4) participants were determined to have experienced BW changes if they showed a 5%–15% increase or reduction in their BW at the end of 12-month follow-up period [28], (5) an informant who knew the participant well must be available, and the patients were asked to have a repeat measurement of their weight within the follow-up tenure of one year. Exclusion criteria were a recent cardiovascular event, a weight gain/loss > 9 kg in the three months before the beginning of the study, the recent use of weight loss medications or medications that caused a weight gain, cancer cachexia that caused unintentional weight loss, or medical conditions that prevented the full participation in the research. Information was collected from a standardized clinical examination and questionnaires. BW was measured and the baseline questionnaires were completed for all the participants, followed by the questionnaires for their diet and lifestyle. All questionnaires were completed and returned at 12 months. Different therapeutic factors can largely affect the individual’s metabolic profiles in the clinical study. To minimize those discrepancies, all the samples were collected in the morning after overnight fasting for all participants included in this study. Each participant’s or patient’s record was linked to their personal identification number, which is unique to every citizen in China, enabling the linkage of clinical data from the first and the second check-up for each individual. Consequently, 46 subjects comprised the cohort for this study (26 males and 20 females). Of them, 24 were N–H subjects (12 males and 12 females) and 22 were H–N subjects (14 males and 8 females).

### Standardized measurements

During both the annual check-ups, the participants’ height was measured to the nearest centimetre without shoes, and the BW was measured to the nearest half kilogram (kg) wearing light clothes without shoes. Similar methods were applied in both the health check-ups. BMI was calculated by dividing a participant’s BW in kg by the squared value of the body height in metres (kg/m^2^). The changes in the BMI between health check-ups were categorized into two types as N–H and H-N. The N–H BMI was considered 18.5 ≤ BMI < 24 kg/m^2^ initially and BMI ≥ 24 kg/m^2^ next year), and H-N BMI was defined as the BMI at the first health check-up greater than or equal to 24 kg/m^2^ and 18.5 ≤ BMI < 24 kg/m^2^ next year, according to the Chinese Obesity Working Group and World Health Organization categorization. At both health check-ups, each participant’s blood pressure (BP) was measured by specially trained nurses or technicians with the participant sitting with their arm resting on a table at heart level. BP was measured using a Yuyue YE8700A (Yuyue, China). HTN was defined as systolic blood pressure (SBP) ≥ 140 mmHg and/or diastolic blood pressure (DBP) ≥ 90 mmHg [29, 30]. We calculated the change in the SBP and the DBP between health check-ups for each individual by subtracting the SBP and the DBP at the first health check-up from the SBP and the DBP at the second health check-up.

### Blood sampling

Fasting blood samples were drawn from the participants via venipuncture by nurses. It was immediately centrifuged for 10 min at 3000 g at 4 °C to obtain the serum. 200 μl of methanol was added to 100 μl serum. The mixture was vortexed for 30 s. Then the samples were centrifuged for 15 min at 4 °C. Subsequently, the supernatant was collected and stored at − 80 °C.

### Metabolic profiling

Liquid chromatography quadrupole-time-of-flight mass spectrometer (LC-Q-TOF–MS) analysis is based on our team’s common metabolic profiling protocol [31]. An LC-Q-TOF–MS analysis was performed with an Agilent 1200 Series LC Systems, Agilent technologies electrospray ionization and Agilent 6520 Accurate-Mass Q-TO. Separation of all of the samples was performed on Eclipse plus C18 (3.6 mm × 100 mm, 1.8 µm) with column temperature 45 °C. Mass spectrometer detection was operated in the positive ion mode with the required settings [31]. All analyses were acquired by the instrument mass spray to improve reproducibility and accuracy. Leucine Enkephalin has been used as reference material for MS tuning and calibration at a concentration of 50 fmol/µl. The data for each sample were normalized to a total area. Partial least squares discriminant analysis (PLS-DA) was performed for the metabolic profile analysis. The differentiation performance was validated by the area under the curve (AUC) of receiver operating characteristic curves. A multivariate analysis was done with the SIMCA-P software (Umetrics AB, Sweden) and SAS (v9.2, SAS Institute, Inc., Cary, NC.). A Chi-square test was performed for the analysis of the attribute data. The variance analysis was used for comparisons between the groups. A *p*-value of less than 0.05 was considered statistically significant.

Representative value (RV) is defined as the power of the metabolite to reflect the abnormality of the disease [31]. RV uses the fold change of the number of enzymes catalysing those metabolites and the importance of every enzyme to evaluate the indicative ability of the metabolite for the diseases.

$${\mathrm{R}\mathrm{V}}_{m}=\frac{{FC}_{m}\sum _{i=1}^{{n}_{e}}{EP}_{i}}{\sum _{j=1}^{{n}_{m}}{(FC}_{{m}_{j}}\sum _{i=1}^{{n}_{e}}{EP}_{i})}$$

${\mathrm{R}\mathrm{V}}_{\mathrm{m}}$: the representative value for metabolite, m;

${\mathrm{E}\mathrm{P}}_{\mathrm{i}}$: the network power of enzyme $\mathrm{i}$ that participates catalyzing metabolite, m;

${\mathrm{n}}_{\mathrm{e}}$: the number of enzymes that participates in catalysing metabolite, m;

${\mathrm{F}\mathrm{C}}_{\mathrm{m}}$: the fold change value of metabolite, m;

${\mathrm{n}}_{\mathrm{m}}$: the number of deregulated metabolites.

### Protein profiling

The matched samples were crushed, dissolved in lysis buffer (7 M urea, 2 M thiourea, 4% CHAPS, 0.5% IPG buffer, 3–10 NL, 0.5% Triton) containing protease inhibitor mixture. Protein identification and 2D gel separation were then carried out. The peptides were generated by trypsin. MS/MS spectra were processed by Analysis Software (v2.0, Applied Biosystems™) and searched using Mascot (v2.4, Matrix Science). The resulting MS/MS spectra were searched against the MS protein sequence database. Student’s *t *tests, two-sided were used to analyze differences in protein levels.

### Ingenuity pathway analysis (IPA)

The analyses of canonical pathways, bio-functions and the networks were conducted by using the Ingenuity Pathway Analysis system (IPA, http://www.ingenuity.com) for the candidate metabolites and proteins to gain further insights into the typical metabolic alterations.

## Results

### Baseline characteristics of participants

A total of 46 patients were included. Their ages ranged from 41 to 76 years (N–H 62.8 ± 8.3 vs. H–N 64.2 ± 11.9). Cases in men were in the majority (56.5%). After one-year follow up, BMI change from 24.3 ± 0.5 to 25.7 ± 0.7 in N–H group and 26.1 ± 1.4 to 23.8 ± 0.9 in H-N group, respectively. No clinically relevant differences were observed between two groups by different factors such as exercises, eating habits, dairy foods, fruits, vegetables, beverage, meat, sweets, alcohol, smoking influencing both the BW and metabolic changes potentially. The demographic and baseline clinical characteristics of the enrolled participants are shown in Table 1.

**Table 1:** Baseline characteristics and clinical measurements of 46 participants during the follow-up

Variables	BMI change categorization	
	N–H (n = 24)	H–N (n = 22)
Age (y)	62.8 ± 8.3	64.2 ± 11.9
Men (%)	12 (50.0%)	14 (63.7%)
Race or ethnic group		
Han	24	22
Others	0	0
BMI 0 (kg/m2)	24.3 ± 0.5	26.1 ± 1.4
BMI 1 (kg/m2)	25.7 ± 0.7	23.8 ± 0.9
Waist 0 (cm)	85.50 ± 5.16	89.48 ± 6.96
Waist 1 (cm)	86.75 ± 6.09	87.91 ± 6.87
Glucose 0 (mmol/L)	5.77 ± 0.47	5.87 ± 0.50
Glucose 1 (mmol/L)	5.65 ± 0.41	5.69 ± 0.34
Systolic blood pressure 0 (mm Hg)	146.38 ± 16.24	147.10 ± 16.45
Systolic blood pressure 1 (mm Hg)	148.59 ± 16.02	146.05 ± 17.64
Diastolic blood pressure 0 (mm Hg)	85.50 ± 5.16	89.48 ± 6.96
Diastolic blood pressure 1 (mm Hg)	86.55 ± 5.90	87.71 ± 8.36
Total cholesterol 0 (mmol/L)	5.48 ± 1.11	5.26 ± 1.49
Total cholesterol 1 (mmol/L)	5.33 ± 0.83	4.77 ± 0.94
Triglyceride 0 (mmol/L)	1.65 ± 0.82	1.95 ± 1.32
Triglyceride 1 (mmol/L)	1.66 ± 0.76	1.70 ± 1.26
Tobacco consumption Packs per year, mean ± SD	34.61 ± 19.33	29.87 ± 19.54
Regular cigarette smoking		
Yes	7	5
No	17	16
Unknown	0	1
Regular alcohol consumption		
Yes	5	3
No	18	19
Unknown	1	0
Regular physical activity		
Yes	9	11
No	15	11
Unknown	0	0
Sodium intake		
≤ 6 g/day	5	6
> 6 g/day	15	14
Unknown	4	2
Food groups (no. of servings/day)		
Fruits and juices	1.2	1.3
Vegetables	1.1	1.4
Grains	1.8	1.6
Regular-fat dairy	2.1	2.6
Nuts, seeds, and legumes	1.8	1.9
Beef, pork	1.2	1.1
Poultry	0.9	1.0
Fish	0.3	0.5
Snacks and sweets	1.1	0.9

Data are mean S.E

1 pack per year is equal to smoking 1 pack per year

*BMI* body mass index, *0* baseline , *1*  after 1 year follow up

### Identification of metabolites and proteins

Ten metabolites were identified in the N–H HTN-PDS subjects, and 14 metabolites were identified in the H-N HTN-PDS subjects (Table 2). The cut-off for the fold change of each metabolite and the ratio of each protein in the HTN-PDS patients to those in the control group was set to greater than 1.01-fold, and the FDR p-value as p < 0.01. Three types of PCs were found to get altered. PC (22:2(13Z,16Z)/24:1(15Z)), LysoPC (16:1(9Z)) were decreased in N–H samples, whereas the levels of PC (14:0/16:0) were increased in H-N samples. The results also indicated the levels of phosphatidylinositol, PI(16:0/20:4(5Z,8Z,11Z,14Z)), all-trans-Decaprenyl diphosphate were significantly increased in the weight gain samples. Besides, the levels of 5-amino-1-(5-phospho-D-ribosyl) imidazole-4-carboxylate,3b,5a,6b-Cholestanetriol, 2-aminomuconic acid semialdehyde were significantly decreased in the BW loss samples. A phospho-antibody microarray was used to make a list of proteins whose phosphorylation states were increased or decreased in the HTN-PDS patients, and 45 phosphorylated proteins in the HTN-PDS patients were found to be altered, with 23 up-regulated and 22 down-regulated (Table 3).

**Table 2:** Metabolites detected by LC-Q-TOF–MS in the validation sample set (HTN-PDS patients with body mass index from normal to high (N–H))/high to normal (H-N)) and their biological variation

Rt	Mass	Name	Formula	Folder	RV
Hypertensive patients with BMI from normal to high (N–H)					
883.853	923.7343	PC(22:2(13Z,16Z)/24:1(15Z))	C54H102NO8P	− 4.2589	0.30
488.784	493.3168	LysoPC(16:1(9Z))	C24H48NO7P	− 1.3129	0.20
502.977	520.2719	Dolichyl diphosphate	C25H46O7P2	1.1990	0.10
515.839	761.4996	PE(16:1(9Z)/22:6(4Z,7Z,10Z,13Z,16Z,19Z))	C43H72NO8P	1.7277	0.10
275.788	491.0008	dATP	C10H16N5O12P3	− 1.3376	0.08
863.462	858.5258	PI(16:0/20:4(5Z,8Z,11Z,14Z))	C45H79O13P	5.7789	0.06
868.575	858.5692	All-trans-Decaprenyl diphosphate	C50H84O7P2	5.7789	0.02
256.068	432.066	Se-Adenosylselenohomocysteine	C14H20N6O5Se	1.0574	0.02
1170.565	412.0185	dIDP	C10H14N4O10P2	− 2.4528	0.01
277.447	482.9845	Cytidine triphosphate (CTP)	C9H16N3O14P3	1.2429	0.01
Hypertensive patients with BMI from high to normal (H-N)					
1141.523	705.5309	PC(14:0/16:0)	C38H76NO8P	6.4684	0.32
652.833	420.3603	3b,5a,6b-Cholestanetriol	C27H48O3	− 7.4571	0.16
521.412	722.444	Octaprenyl diphosphate	C40H68O7P2	2.4689	0.12
190.274	141.0426	2-aminomuconic acid semialdehyde	C6H7NO3	− 4.6339	0.10
61.626	129.0426	Pyroglutamic acid	C5H7NO3	6.5815	0.08
223.019	189.0637	N-Acetyl-L-glutamic acid	C7H11NO5	3.2199	0.06
324.918	462.2618	Retinyl beta-glucuronide	C26H38O7	4.5571	0.05
89.597	168.0283	Uric acid	C5H4N4O3	− 1.7995	0.03
864.632	130.063	3-Methyl-2-oxovaleric acid	C6H10O3	3.3563	0.02
190.188	175.0633	3-Indoleacetic Acid	C10H9NO2	− 1.0880	0.02
66.389	190.0114	Oxalosuccinic acid	C6H6O7	− 3.0817	0.02
51.332	339.0468	5-amino-1-(5-phospho-D-ribosyl)imidazole-4-carboxylate	C9H14N3O9P	− 13.5318	0.01
63.879	112.016	2-Furoic acid	C5H4O3	− 2.4225	0.01
625.18	362.2093	18-Hydroxycorticosterone	C21H30O5	3.9819	0.01

“Folder” refers to the “normal BMI vs. high BMI” change value; RV is the power of the metabolite to reflect the abnormal state in the disease

LysoPC: Lysophosphatidylcholine; PC: Phosphatidylcholine

**Table 3:** Identified proteins in HTN-PDS patients with body mass index (BMI) from normal to high (N–H)

ANOVA (p)	Folder	Protein ID	Gene name	Score	No. of peptide identified
Hypertensive patients with BMI from normal to high (N–H)					
0.011	+ 2.3	Apolipoprotein A–I	APOA1	105.00	2
0.010	+ 2.2	Angiotensinogen	AGT	316.00	3
0.013	+ 2.1	Apolipoprotein D	APOD	41.00	2
0.019	+ 2.1	Serum amyloid P-component	APCS	176.00	3
0.014	+ 1.6	Apolipoprotein E	APOE	300.00	7
0.028	− 1.5	Haptoglobin-related protein	HPR	42.00	3
0.033	− 1.5	Alpha-2-macroglobulin	A2M	216.00	4
0.035	− 1.5	Mannan-binding lectin serine protease 2	MASP2	36.00	1
0.007	− 1.6	Antithrombin-III	SERPINC1	92.00	3
0.042	− 1.6	Keratin, type I cytoskeletal 14	KRT14	51.00	2
0.004	− 1.7	Complement factor I	CFI	116.00	5
0.044	− 1.7	Complement factor H-related protein 1	CFHR1	117.00	3
6.14E-05	− 1.8	Plasma serine protease inhibitor	SERPINA5	141.00	3
0.003	− 1.8	Pigment epithelium-derived factor	SERPINF1	907.00	9
0.034	− 1.8	ELAV-like protein 3	ELAVL3	37.00	1
0.038	− 1.8	Apolipoprotein L1	APOL1	221.00	4
0.047	− 1.8	Keratin, type I cytoskeletal 10	KRT10	208.00	4
0.037	− 2.0	Mitochondrial coenzyme A transporter	SLC25A42	35.00	1
0.039	− 2.0	Haptoglobin	HP	118.00	3
0.042	− 2.0	Apolipoprotein C-III	APOC3	42.00	1
0.022	− 2.5	Hepatocyte growth factor-like protein	MST1	99.00	4
0.014	− 2.7	Complement C3	C3	126.00	3
0.004	− 6.5	Complement C4-A	C4A	106.00	4
Hypertensive patients with BMI from high to normal (H–N)					
1.02E-05	+ 8.8	Prothrombin	F2	206.00	2
0.030	+ 3.8	Serum amyloid A-4 protein	SAA4	428.00	3
1.41E-04	+ 3.2	Hemopexin	HPX	123.00	3
0.014	+ 2.9	Complement factor H-related protein 1	CFHR1	722.00	4
0.013	+ 2.5	Complement component C6	C6	480.00	3
0.001	+ 2.5	Complement C3	C3	290.00	5
0.023	+ 2.4	Fibrinogen alpha chain	FGA	58.00	1
0.038	+ 2.0	Beta-2-glycoprotein 1	APOH	152.00	3
0.006	+ 1.8	Apolipoprotein E	APOE	134.00	2
0.019	+ 1.7	Insulin-like growth factor II	IGF2	94.00	2
0.011	+ 1.7	Keratin, type II cytoskeletal 1	KRT1	315.00	7
0.005	+ 1.7	Pigment epithelium-derived factor	SERPINF1	192.00	5
0.011	+ 1.6	Alpha-2-macroglobulin	A2M	318.00	4
0.010	+ 1.6	Plasminogen	PLG	58.00	2
0.011	+ 1.5	Complement C4-A	C4A	745.00	3
0.010	+ 1.5	Inter-alpha-trypsin inhibitor heavy chain H1	ITIH1	188.00	1
0.003	+ 1.5	Apolipoprotein A-IV	APOA4	198.00	6
1.84E-04	+ 1.5	Keratin, type I cytoskeletal 10	KRT10	1037.00	13
0.019	− 1.5	Complement factor I	CFI	69.00	1
0.045	− 1.6	N-acetylmuramoyl-L-alanine amidase	PGLYRP2	142.00	3
0.014	− 1.7	Apolipoprotein L1	APOL1	365.00	6
0.038	− 1.8	Apolipoprotein C-III	APOC3	126.00	1

“Folder” refers to the “high BMI vs. normal BMI” change value

### Biological pathway analysis

**Fig. 1:**
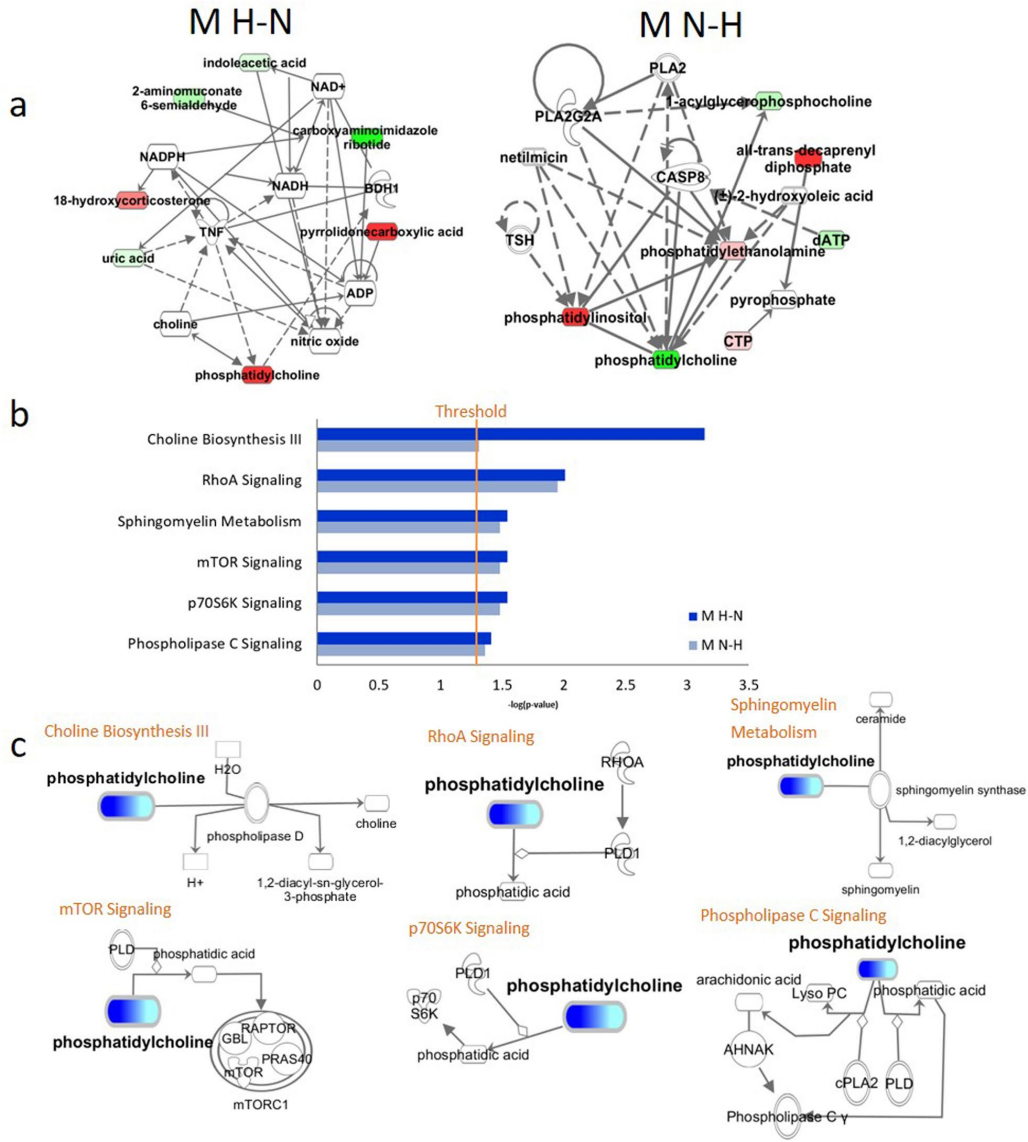
Biological network and canonical pathways related to the identified metabolites in Hypertensive Patients with Phlegm-Dampness Syndrome. **A** Ingenuity Pathway Analysis (IPA)-identified H–N/N–H metabolites network. The N–H BMI was considered 18.5 ≤ BMI < 24 kg/m^2^ initially and BMI ≥ 24 kg/m^2^ next year), and H-N BMI was defined as the BMI at the first health check-up greater than or equal to 24 kg/m^2^ and 18.5 ≤ BMI < 24 kg/m^2^ next year, according to the Chinese Obesity Working Group and World Health Organization categorization. Molecules in the network are represented as nodes, and the biological relationship between two nodes is represented as a line. The coloured symbols represent the metabolites, proteins and pathways that occurred in the findings, while the transparent entries are molecules from the Ingenuity Knowledge Database. Red symbols represent up-regulated metabolites or proteins, the light red symbols represent the modestly up-regulated metabolites/proteins, green symbols represent down-regulated metabolites/proteins, and the light green symbols represent the modestly down-regulated metabolites/proteins. Solid lines between molecules indicate a direct physical relationship between the molecules, while dotted lines indicate indirect functional relationships. **B** IPA identified six biological pathways. The Y-axis represents the top functions as calculated by IPA based on differentially expressed genes, and the X-axis represents the ratio of a number of genes from the dataset that map to the pathway and the number of all known genes ascribed to the pathway. The orange threshold line marks the p = 0.05. The threshold of p-value < 0.05 as calculated by Fischer's test. **C** IPA identified most highly rated network. The network representation of the most highly rated network. Phosphatidylcholine (PC) shaded dark blue are upregulated and those that are light blue are downregulated. A solid line represents a direct interaction between the two metabolites

**Fig. 2:**
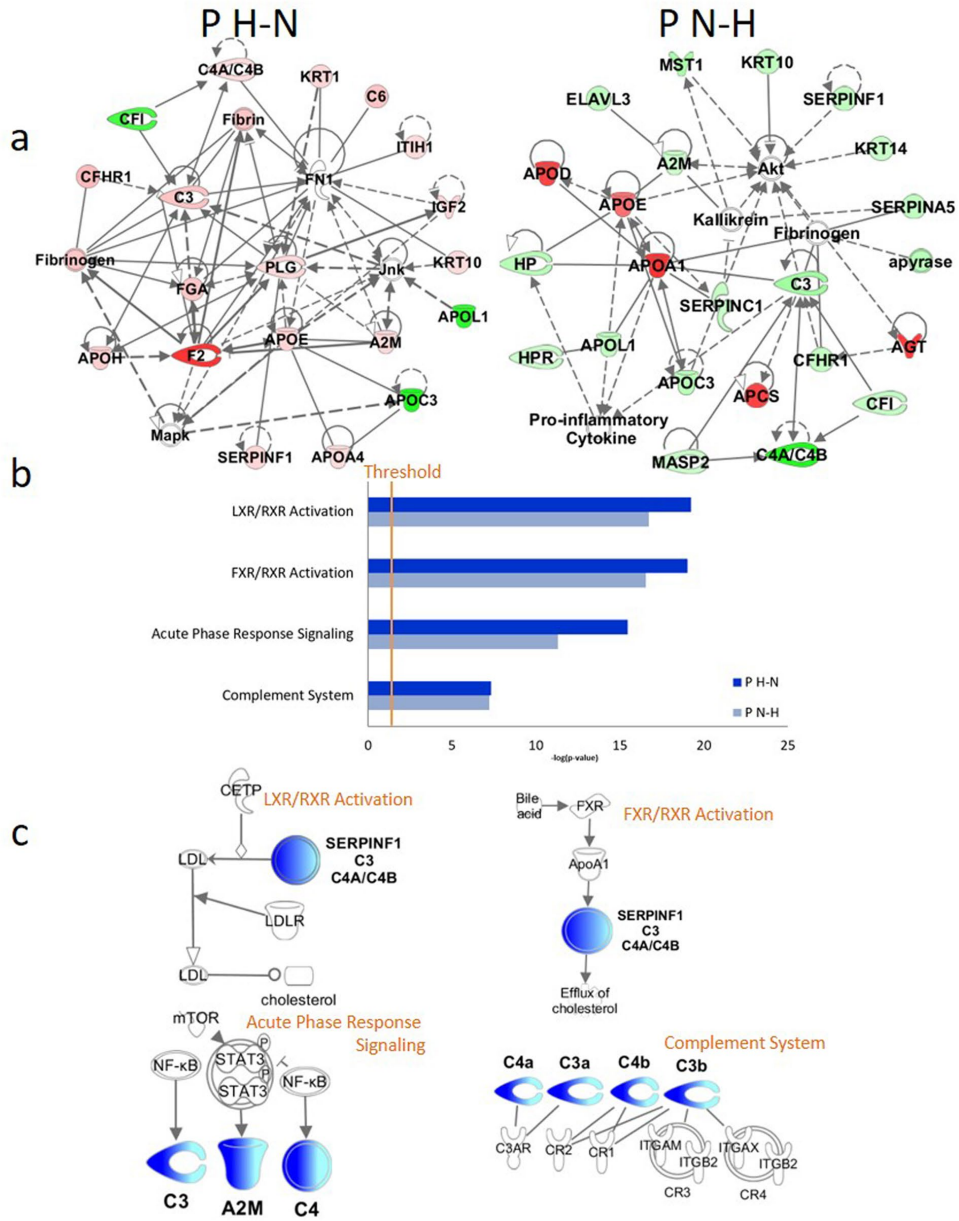
Biological network and canonical pathways related to the identified proteins in Hypertensive Patients with Phlegm-Dampness Syndrome. **A** Ingenuity Pathway Analysis (IPA)-identified H–N/N–H proteins network. The N–H BMI was considered 18.5 ≤ BMI < 24 kg/m^2^ initially and BMI ≥ 24 kg/m^2^ next year), and H-N BMI was defined as the BMI at the first health check-up greater than or equal to 24 kg/m^2^ and 18.5 ≤ BMI < 24 kg/m^2^ next year, according to the Chinese Obesity Working Group and World Health Organization categorization. Molecules in the network are represented as nodes, and the biological relationship between two nodes is represented as a line. The coloured symbols represent the metabolites, proteins and pathways that occurred in the findings, while the transparent entries are molecules from the Ingenuity Knowledge Database. Red symbols represent up-regulated metabolites or proteins, the light red symbols represent the modestly up-regulated metabolites/proteins, green symbols represent down-regulated metabolites/proteins, and the light green symbols represent the modestly down-regulated metabolites/proteins. Solid lines between molecules indicate a direct physical relationship between the molecules, while dotted lines indicate indirect functional relationships. **B** IPA identified pathway: LXR/RXR activation, FXR/RXR activation, acute phase response signalling and the complement system. The Y-axis represents the top functions as calculated by IPA based on differentially expressed genes are highlighted and the X-axis represents the ratio of the number of genes from the dataset that map to the pathway and the number of all known genes ascribed to the pathway. The orange threshold line marks the p = 0.05. The threshold of p-value < 0.05 as calculated by Fischer's test. **C** IPA identified most highly rated network. The network representation of the most highly rated network. Complement C3, Complement C4a/C4b, Alpha-2-macroglobulin (A2M), Serpin family F member 1 (SERPINF1) that are shaded were determined to be significant from the statistical analysis. These proteins shaded dark blue are upregulated and those that are light blue are downregulated. A solid line represents a direct interaction between the two proteins

**Fig. 3:**
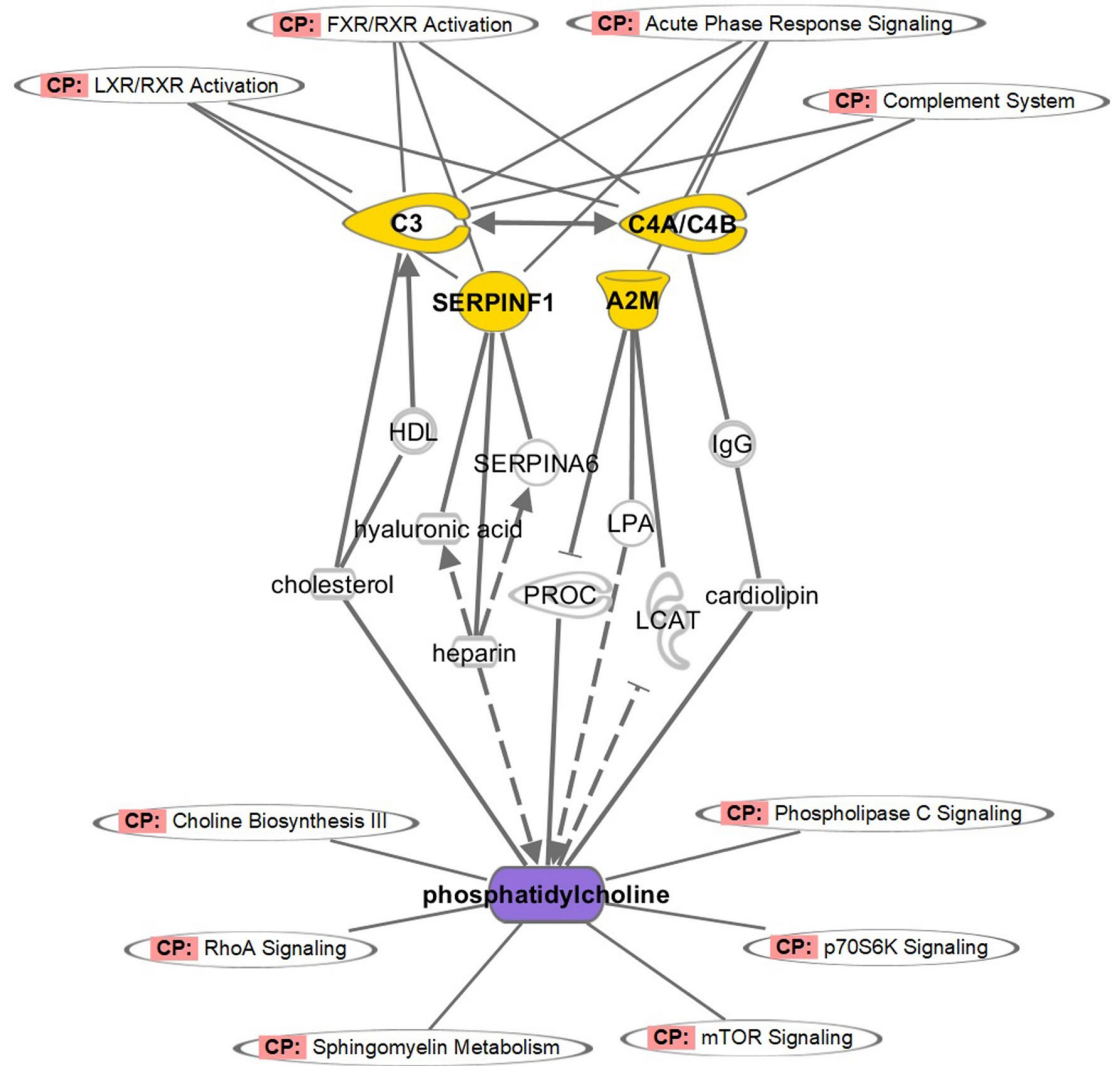
Network analysis identifies metabolites and protein clusters of functional importance in Hypertensive Patients with Phlegm-Dampness Syndrome. The reconstructed network contains, Complement C3, Complement C4a/C4b, A2M, SERPINF1 (yellow) and PC (purple). Edges (lines and arrows between nodes) represent direct (solid lines) and indirect (dashed lines) interactions between molecules as supported by information in the Ingenuity knowledge base. Node shapes represent functional classes of gene products, rectangles with solid lines for cytokines, rectangles with dotted lines for growth factors, triangles for phosphatases, concentric circles for groups or complexes, diamonds for enzymes, and ovals for transcriptional regulators or modulators

Biological association network and pathways related to a series of identified metabolites were identified using IPA software. Using this we were able to reconstruct the metabolite networks and proteins networks for H-N/N–H groups of participants. Those reconstructed networks were generated using the fusion of proteomics and metabolomics data. They helped to identify a series of metabolites and proteins associated with several key biological processes and signalling pathways. Firstly, focusing with the reconstructed metabolites networks, 28 prominent nodes were identified related to the biological functions including but not limited to choline biosynthesis, sphingomyelin metabolism. Our analysis returned with some knowledge on metabolites (such as indoleacetic acid, uric acid, carboxyaminoimidazole in H-N network, and phosphatidylcholine, phosphatidylinositol, CTP in N–H network), proteins (such as APOL1, APOC3 in H–N networks, and APOD, APOA1 in N–H network) and their associations with the pathways like choline biosynthesis, mTOR signalling, sphingomyelin metabolism. Their dependencies to each other have been depicted in Fig. 1A. This also indicated a wide range of metabolites those were up-regulated and down-regulated. For example, the metabolites such as phosphatidylcholine, 18-hydroxycorticosterone, pyrrolidonecarboxylic acid were found to be upregulated in H–N cohort and phosphatidylinositol, trans-decaprenyl diphosphate in N–H cohort. On the other side, the metabolites such as indoleacetic acid, uric acid, carboxyaminoimidazole were found to be down-regulated in H–N cohort and phosphatidylcholine, acyl-glycerophosphocholine in N–H cohort (Fig. 1A). The physical connections, as well as the functional implications among them, were established. Next, we were able to identify six important biological pathways out of those changes in the level of the metabolites. They were choline biosynthesis III, RhoA signalling, sphingomyelin metabolism, mTOR signalling, p70S6k signalling and phospholipases C signalling (Fig. 1B). Among them, the Phosphatidylcholine (PC) signalling pathway was determined to be the most significant (Fig. 1C). Secondly, the H–N/N–H proteins network analysis, 45 prominent nodes were identified related to the biological functions such as LXR/RXR activation, FXR/RXR activation, complement system activation. Our analysis returned with some knowledge on metabolites, proteins (as described above) and their associations with the pathways like LXR/RXR and FXR/RXR activation, acute phase response signalling, complement system activation. Their dependencies to each other have been depicted in Fig. 3A. This finding indicated a wide range of proteins beings up-regulated and down-regulated. For example, C3, F2, SERPINF1 were found to be up-regulated in H-N network, and APOD, APOE, APOA1 in N–H networks. And APOC3, APOL1, CF1 were found to be down-regulated in H-N network, wherein MST1, APOL1, SERPINC1 got down-regulated in N–H network (Fig. 2A). Then, we investigated the LXR/RXR activation, FXR/RXR activation, acute phase response signalling and complement system successively. It indicated several important proteins associated with the biological functions of those pathways (Fig. 2B). Of them, Complement C3, Complement C4a/C4b, Alpha-2-macroglobulin (A2M), Serpin family F member 1 (SERPINF1) were found to be most significant (Fig. 2C).

### A fusion of insights obtained from metabolomics and proteomics study

To interpret the data obtained from metabolomics and proteomics study, we constructed a correlation network that embodied the interconnections between the variations among proteins and metabolites. We performed the analysis of the networks comprising of Complement C3, Complement C4a/C4b, A2M, SERPINF1, and PC. Interaction between C3 and C4A/C4B were found to be direct, wherein the interaction between LPA and phosphatidylcholine were identified as indirect. All detailed direct and indirect interactions along with their functional classes have been depicted in Fig. 3. Our results revealed that the primary disturbed pathways in response to Phosphatidylcholine metabolism were choline biosynthesis III, RhoA signalling, sphingomyelin metabolism, mTOR signalling, p70S6k signalling and phospholipases C signalling. Besides, we also identified the most relevant pathway of the complement system includes LXR/RXR activation, FXR/RXR activation, and acute phase response signalling. This interaction network offered a global view of potentially functional relationships between metabolites, connected proteins, and pathways.

## Discussions

Using proteomics and metabolomics together, our study strongly endorses the strongest predictors and the associated signalling pathways that are functionally related to longitudinal weight changes of HTN-PDS. It also claims a prominent correlation between body weight and HTN-PDS. Our approach is a pioneer in its class using such approaches together to establish PC, Complement C3, C4a/C4b, A2M and SERPINF1 as markers for HTN control from a longitudinal study. Metabolomic data and proteomic data, those two major directions will be further discussed in detail.

### Phosphatidylcholines as signature metabolic markers for HTN-PDS patients

The metabolomic study has identified various types of PCs getting altered in HTN-PDS patients. Interestingly,, three candidates, PC(22:2(13Z,16Z)/24:1(15Z)), LysoPC(16:1(9Z)) and PC(14:0/16:0) were characterized to exert the strongest impact in discriminating H-N/N–H subjects (Fig. 2C). PCs are a type of glycerophospholipid containing the polar phosphocholine at the head and two nonpolar fatty acid hydrocarbon chains as tails. They are the major membrane-forming phospholipid in mammalian cells [32, 33]. With the accumulation of fat molecules, the concentration of PCs used to get altered. Significantly higher abundance of the PCs was found in overweight compared to normal subjects (N–H: − 4.2589; H–N: 6.4684). This signifies PCs as potential indicators for differential states of metabolic activity between normal and overweight HTN-PDS patients.

Although HTN is closely linked to dyslipidaemia [34], there are quite a limited number of cohorts exists that investigated blood parameters in PC metabolism concerning HTN [35]. A study with the US-Hispanic population elucidated the associations of PCs with HTN using serum-derived lipidomic profiling [36]. Moreover, knowledge suggests that the most PCs are formed by the choline pathway, and in our study, we have attempted to understand the underlying relationships of the lipid, blood pressure nexus with weight change with longitudinal evidence. Our results showed a coherent consistency with the existing benchmark studies while establishing the functional associations with the identified biological pathways and interpreting their terms. Previous studies indicated the elevated levels of sphingomyelin and PC are significantly interrelated in patients with HTN [37]. Sphingomyelin metabolism plays critically in communicating between cells and their environments, being involved in signal transduction and metabolite exchange [38]. Our results indicated that the mechanisms controlling HTN by weight change are more likely to be linked with enhanced metabolic efficiency. Obesity affects mTOR/p70S6k pathway (Figs. 1C, 3). A report demonstrated that weight loss critically regulates the mTOR/p70S6k pathway by promoting protein synthesis in rat models with obesity [39]. Several studies have examined the role of Rho-kinase (ROCK) signalling in cardiac remodelling associated with obesity [40]. ROCK is a downstream effector molecule of a key signalling factor, Ras homolog gene family member A (RhoA). RhoA is a small GTPase that used to get involved in diverse cellular functions. RhoA signalling through its downstream effector, ROCK has been implicated in the pathogenesis of cardiac fibrosis. RhoA activation has significant effects on various cardiovascular diseases, mainly HTN [41, 42]. In this direction, metabolite profiling helped in identifying some lipid metabolites such as PI (16:0/20:4(5Z, 8Z, 11Z, 14Z)), Pyroglutamic acid and 3b,5a,6b-Cholestanetriol as differentially abundant in the serum of N–H/H–N groups, respectively. A research study detected serum adiponectin (APN) levels in 250 hypertensive patients (137 with PDS and 113 non PDS) by showed significant differences in serum APN levels between PDS and non-PDS patients (5.07 ± 0.35 μg/mL vs. 6.41 ± 0.39 μg/mL, p = 0.045) [43]. Moreover, in CM theory, the same strategy is used to treat patients with multiple diseases who have the same syndrome. Multiple diseases with PDS tend to be obese and have worse clinical outcomes [14]. Our results can be used to optimize personalized treatment for high-risk patients, or be used as key metabolites to follow when regulating body weight.

### Complement system activity

Our proteome analysis revealed a higher abundance of lipoprotein and Complement components. Among those, Complement C3, Complement C4a/C4b, A2M, SERPINF1 have essentially the same tendencies of variation in both H–N and N–H group (Fig. 2A). Evidence suggests that C3 and C4 are both increased in chronic inflammation [44, 45]. A recent large cohort study with 2000 healthy men with an average of 15 years follow-up reported the association of the complement system with HTN pathogenesis [45]. This study has shown that the subjects with a high baseline C3 levels were at a higher risk of developing HTN in later life irrespective of any sort of interference of risk factors. Even at the baseline, the levels of C3 and C4 were found to be well correlated with the blood pressure of the subjects [45]. Within the similar pace, our results also indicated the higher relevance of C3 (N–H: − 2.7; H–N: + 2.5), C4a (N–H: − 6.5; H–N: + 1.5) in the considered obese HTN subjects. The C3 and C4a levels were found to be elevated in our study subjects as well (Fig. 2B, C). Certainly, this indication is quite consistent with the existing established knowledge from the large study [45]. C3 is also being considered as an essential factor in the pathogenesis of HTN due to its maintenance effect on undifferentiated mesenchymal cells. There was enough evidence suggesting the connection between inflammation and HTN, but the specific mechanisms of complement systems were not well explored in HTN pathogenesis [46]. It is indeed a deep area for further investigation to reveal the mechanistic insights and the comprehensive interactions between HTN and inflammation. On the other hand, retinoid X receptor (RXR), that plays an important role in cardiac development and whose impairment is linked to developing cardiovascular diseases [47]. They serve as the major target for a group of transcription factors, Liver X receptor (LXR) α and β belonging to the nuclear receptor (NR) superfamily [47]. They form heterodimers and bind to the RXR. RXR agonists were found to inhibit HTN-induced myocardial hypertrophy by modulating p70S6K signalling pathway [48]. Possible involvement of dysregulated LXR signalling in human obesity has been proposed in several independent studies [49, 50]. Although data from murine models discussed above suggest that LXR have several beneficial effects, including reduced cardiovascular risk related enhanced reverse cholesterol transport, increased glucose uptake, the attenuated release of pro-inflammatory factors, increased lipid oxidation and reduced atherosclerosis, data on the systemic effects of LXR agonists in humans are lacking. The farnesoid X receptor (FXR) is a type of nuclear receptor that functions as an endogenous sensor for bile acids (BAs). Our data has also predicted the activation of FXR/RXR pathway. Upon activation, FXR heterodimerizes with RXR and regulates genes involved in the cholesterol synthesis pathway (Fig. 2C). Cholesterol is one of the strongest inverse predictors for cardiovascular diseases. Data from our study also offered a set of proteins and interactors such as HDL, Cardiolipin, LPA (Fig. 3) those are linked to cardiovascular physiology and their impairments may lead to cardiovascular diseases. Considering the functional evidence endorsing our results, a systematic study identified A2M-related biological processes getting perturbed in the pathophysiology of Alzheimer’s diseases. Besides, the gene, SERPINF1 was found to encode a pigment epithelium-derived factor, a multi-functional protein that exhibits neurotrophic and anti-angiogenic activity [51]. These findings together must lead to powerful support in the view of epidemiological studies in which the changes in body weight has been demonstrated as a risk factor for developing HTN.

The best strength of our study is that the data are longitudinal, and our mode of analysis integrated two cutting-edge methods, metabolomics, and proteomics. It has several prominent advantages in translational clinical science. However, encompassing the maximum number of variables, such as ethnicity, race etc. and their effects on the study subjects were kept out of consideration in this study designs. This is a limitation in this study as well as a small number of participants. They may have a potential impact on the proteome or metabolome levels which were not explored. All study subjects were Han Chinese in our study, and we have strictly defined the criteria to reduce the potential sample bias. Moreover, having a larger dataset, especially more samples of HTN with PDS compare to non PDS, and is warranted to support these preliminary findings. It was also shown that greasy food, lack of exercise, and smoking were all important factors of the PDS, dynamic monitoring these factors is needed in further studies.

## Conclusions

In conclusion, the combined serum proteomic and metabolomic profiling reveals a link between the complement system, altered phosphatidylcholine metabolism and HTN patients with body weight changes. PC, Complement C3, Complement C4a/C4b, A2M and SERPINF1 were identified as the strongest predictors of HTN patients’ weight changes. The present study also clarified critical pathways that function in HTN patients with body weight changes, they are important resource to support HTN biomarker discovery. Future studies with larger cohorts are required to validate these findings.

## Data Availability

The datasets generated and/or analyzed during the current study are available from the corresponding author upon reasonable request.

## Abbreviations

A2M: Alpha-2-macroglobulin
BP: Blood pressure
BMI: Body mass index
DBP: Diastolic blood pressure
FXR: Farnesoid X receptor
HTN: Hypertension
IPA: Ingenuity pathway analysis
Kg: Kilogram
LC-Q-TOF–MS: Liquid chromatography quadrupole-time-of-flight mass spectrometer
LXR: Liver X receptor
NR: Nuclear receptor
PC: Phosphatidylcholine
PDS: Phlegm-Dampness Syndrome
RhoA: Ras homolog gene family member A
RV: Representative value
RXR: Retinoid X receptor
SERPINF1: Serpin family F member 1
